# Basic maternal health care coverage among adolescents in 22 sub-Saharan African countries with high adolescent birth rate

**DOI:** 10.7189/jogh.10.021401

**Published:** 2020-12

**Authors:** Liliana Carvajal, Emily Wilson, Jennifer Harris Requejo, Holly Newby, Cristina de Carvalho Eriksson, Mengjia Liang, Mardieh Dennis, Fatima Gohar, Aline Simen-Kapeu, Priscilla Idele, Agbessi Amouzou

**Affiliations:** 1 Data and Analytics Section, Division of Data, Analytics, Policy and Monitoring, United Nations Children’s Fund UNICEF, HQ, New York, New York, USA; 2 Department of International Health, Johns Hopkins Bloomberg School of Public Health, Baltimore, Maryland, USA; 3 Independent consultant, Stockholm, Sweden; 4 Health Section, Programme Division, UNICEF HQ, New York, New York, USA; 5 Population and Development Branch, Technical Division, United Nations Population Fund (UNFPA), New York, New York, USA; 6 London School of Hygiene and Tropical Medicine LSHTM, London, UK; 7 Regional Office for Eastern and Southern Africa ESARO, UNICEF, Nairobi, Kenya; 8 Regional Office for West and Central Africa, WCARO, UNICEF, Dakar, Senegal; 9 Office of Research-Innocenti, UNICEF Florence, Italy

## Abstract

**Background:**

In the sub-Saharan Africa region, the adolescent birth rate is the highest in the world, estimated at 100.5 births per 1000 women aged 15 to 19 years, and 2.4 times greater than the global average. This analysis examines coverage levels and gaps in basic maternal health care for adolescent mothers living in this region.

**Methods:**

We used data from national Demographic and Health Surveys (DHS) and Multiple Indicator Cluster Surveys (MICS) conducted between 2010 and 2016 in 22 of the sub-Saharan African Countdown to 2030 priority countries with adolescent birth rates above 100 in 2016. We analyzed 11 indicators of coverage of key services provided during the pre-pregnancy, pregnancy, delivery and postnatal period. We described the coverage level among adolescent girls aged 15-19 and women aged 20-49 for basic indicators in the continuum of care. We conducted a multilevel random effect logistic regression to quantify the association between the receipt of basic package of maternal care and woman’s socio-demographic and socio-economic characteristics.

**Results:**

The median coverage of the basic package of maternal care among adolescents was extremely low, at 9.3%. Adolescent mothers who were in the highest household wealth quintile (odds ratio OR = 2.44, 95% confidence interval (CI) = 2.23-2.68), living in an urban area (OR = 1.25, 95% CI = 1.18-1.33) and having secondary education (OR = 1.61, 95% CI = 1.50-1.73) had greater odds of receiving the basic package of maternal health care as compared to those in the lowest wealth quintile, living in rural areas, and with no education respectively. Adolescent girls aged 15-17 and 18-19 had respectively 26% (OR = 0.74, 95% CI = 0.67-0.82) and 9% (OR = 0.91, 95% CI = 0.84-0.98) lower odds of receiving the basic package compared to women 20-49 years old. Child brides had 12% (OR = 0.88, 95% CI = 0.84-0.93) lower odds of receiving the basic package compared to women who were married after the age of 18.

**Conclusion:**

Coverage of basic maternal health care for adolescent mothers is inadequate in the countries with the highest adolescent birth rates in the world. Addressing the reproductive and maternal health needs of adolescents in sub-Saharan Africa is of critical importance, especially given projections that this region will experience the highest increases in adolescent births in the coming decades.

Globally, adolescents between the ages of 15 and 19 years comprise 8% of the population, or about 600 million [1]. Approximately 16 million girls aged 15 to 19 years and two million girls below 15 years of age give birth annually [2].

An estimated 11% of all global births are to adolescents aged 15 to 19 years [3]. Although the global adolescent birth rate decreased by 27% from 56.4 to 41.2 births per 1000 women aged 15-19 years between 2000 and 2020, progress was uneven and large disparities remain between world regions [4]. Adolescent births are concentrated in low and middle income countries, and particularly in sub-Saharan Africa, where over one in four adolescent girls gives birth before reaching 18 years [5]. Estimated at 100.5 births per 1000 women aged 15 to 19 years, the adolescent birth rate in sub-Saharan Africa is the highest in the world and 2.4 times greater than the global average [4].

Adolescent pregnancy and childbirth are linked to poor perinatal health outcomes and potential long-term negative economic and social consequences. Young adolescent mothers are at increased risk of death [3] and pregnancy-related morbidity, such as pre-eclampsia. The risk of adverse health outcomes is highest among young adolescents who give birth before the age of 15 years as compared to non-adolescent mothers [2,6,7]. Adolescent girls who become parents often faced pressure to discontinue their education, which reduces their employment prospects and puts them at greater risk of poverty [2,8,9]. Policies encouraging equal opportunities for educational attainment and for employment opportunities would likely help decrease the prevalence of adolescent pregnancy [10].

The negative social consequences of adolescent childbirth are often the same factors that place girls at increased risk of early and unintended pregnancy. Girls living in communities that are poorer, less educated, or rural are more likely to become adolescent mothers [11]. And these girls also experience greater challenges with accessing high quality maternal health services compared to their wealthier counterparts [12,13]. Adolescents face multiple barriers in accessing health care and information [14]. In many parts of the world, stigma associated with adolescent pregnancy, health services that are not responsive to adolescent needs, and low financial protection for adolescent mothers may contribute to insufficient coverage of essential maternal health services among this age group [15]. Although recent studies have shown that adolescent girls in low- and middle-income countries have a high unmet need for contraceptive services and often receive low-quality care, comparatively fewer studies have comprehensively examined the coverage, continuity, or quality of maternal care that adolescents receive once they become pregnant [16-19].

The World Health Organization(WHO) now recommends that all pregnant women receive a minimum of eight antenatal care (ANC) contacts, with the first contact occurring within the first three months of pregnancy; have a skilled attendant at birth; and receive routine postnatal health checks by a health provider within the first 48 hours of delivery [20,21]. Prior to 2016, WHO recommended that pregnant women receive a minimum of four visits [20]. Existing research on maternal health service use among adolescents in sub-Saharan Africa suggests that there is wide variation between countries; however, coverage tends to be insufficient and lower among adolescents than among older women. A study of antenatal care use in 13 West African countries found that only 62% of adolescent mothers aged 10-19 years received four or more antenatal visits for their first birth compared to 71% and 81% of women aged 20 to 24 years and 25 to 49 years, respectively [22]. Further, the study found that even among women who received at least four visits, adolescent mothers were less likely to have received recommended interventions for ANC such as blood pressure measurements, and urine and blood testing, and discussing potential pregnancy complications [22]. Similarly, an earlier study of 21 countries in sub-Saharan Africa found that adolescents aged 15-19 years were less likely to receive four or more antenatal visits, deliver in a health facility, and have a skilled birth attendant compared to women aged 20 years and older [23].

Given that sub-Saharan Africa is projected to experience the highest increase in the number of adolescent births between 2010 and 2030, it is imperative to better understand maternal health service use patterns among adolescents in this region and identify areas for programmatic action [11]. Low levels of coverage for maternal health services regardless of age highlight the need to dramatically improve outreach and service delivery overall in sub-Saharan Africa. Adolescents have unique needs which must be taken into account when designing and scaling up adolescent responsive programmes. The aim of this study is to describe coverage of key reproductive and maternal health indicators among adolescent mothers in 22 countries in sub-Saharan Africa with high adolescent birth rates and examine the influence of socioeconomic factors of receiving basic maternal health care.

## METHODS

### Data and study population

This study uses data from 22 countries in sub-Saharan Africa. These countries are ‘Countdown to 2030’ priority countries with high adolescent birth rates, defined as closest to 100 adolescent births per 1000 women aged 15 to 19 years in 2016, given that this is the highest regional average across SDG regions. Among the 25 countries meeting this criterion, 22 countries with a publicly available DHS or MICS conducted since 2010 were included in this analysis and all are in Sub-Saharan Africa (**Table 1**). Data used in these analyses included 18 Demographic and Health Surveys (DHS) and 4 Multiple Indicator Cluster Surveys (MICS) conducted in the period 2010-2016. The DHS and MICS are nationally representative, cross-sectional surveys using multi-stage cluster sampling. In each sampled household, individual questionnaires are administered to all women aged 15 to 49 years covering a range of topics including reproductive health, child health, nutrition, malaria, HIV/AIDS, etc. Both surveys use standardized questionnaires allowing for the analysis of outcomes across countries and surveys. In cases where more than one survey was conducted since 2010, the most recent survey was selected. The total sample comprised 22 135 adolescent girls aged 15-19 years in the households sampled across the 22 countries included in the analysis, with the largest sample in Nigeria (2053 adolescents) and the lowest in Zimbabwe (508 adolescents) (**Table 1**). We examined service coverage levels of all adolescent mothers who reported having at least one live birth in the two years preceding the survey.

**Table 1:** Characteristics of countries and data sets used in analysis*

Country	Number of adolescents (15-19 age group) in the sample	Age specific fertility rate: 15-19 age group	Survey year	Survey type
Angola	1399	163	2015-2016	DHS
Burkina Faso	966	130	2010	DHS
Cameroon	566	128	2014	MICS
Central African Republic	595	229	2010	MICS
Chad	1472	179	2014-2015	DHS
Congo-Brazzaville	884	147	2011-2012	DHS
Côte d’Ivoire	581	129	2011-2012	DHS
Democratic Republic of Congo	1432	138	2013-2014	DHS
Gabon	595	114	2012	DHS
Guinea	649	146	2012	DHS
Guinea-Bissau	579	137	2014	MICS
Kenya	1311	96	2014	DHS
Liberia	720	149	2013	DHS
Malawi	1571	136	2015-2016	DHS
Mali	1333	178	2015	MICS
Mozambique	1081	167	2011	DHS
Niger	836	206	2012	DHS
Nigeria	2053	122	2013	DHS
Sierra Leone	1058	125	2013	DHS
Tanzania	841	132	2015-2016	DHS
Zambia	1105	141	2013-2014	DHS
Zimbabwe	508	110	2015	DHS
Total	22 135			

*Source: Survey data from Demographic and Health Surveys (DHS) and Multiple Indicators Cluster Surveys (MICS).

### Indicators and definitions

This study explored an initial set of 11 indicators related to intervention coverage and content of care for basic maternal health care: demand for family planning satisfied with modern methods, antenatal care contacts (first visit by 3rd trimester, at least four visits, and at least 8 visits); antenatal care content (receipt of all four selected interventions during ANC visits: blood pressure measured, having a blood sample taken, having a urine sample taken, and receiving tetanus toxoid), skilled birth attendant, institutional delivery, staying in health facility at least 24 hours after delivery, postnatal care visits for mother and baby and early initiation of breastfeeding. Descriptive results are presented in Table S1 of the **Online Supplementary Document**. The selection of these indicators was based on WHO recommendations for the antenatal, delivery and postnatal period [19,20,24].

We defined the co-coverage of selected maternal and newborn health interventions that we referred to as basic package of maternal health care. Indicators included are related to service contact or interventions received during antenatal, delivery and postpartum period, based on WHO recommendations for care during these periods [21-23] and data availability. Thus, the basic package of maternal health care consists of the co-coverage of four indicators: having at least four antenatal care contacts, having received 4 key antenatal care content interventions (blood pressure measured, blood and urine sample taken, received tetanus toxoid injection), having had a skilled attendant at birth, and initiating breastfeeding within one hour of delivery. A woman received the basic maternal package when she received all four interventions or services contacts. Other indicators initially assessed in the descriptive part of the analysis were dropped from the basic package due to lack of data across all countries in the analysis. Although early initiation of breastfeeding is specific to newborns, we used it as a proxy for maternal intrapartum care given the measurement issues and data availability for these indicators. A total of 3399 had missing data to accurately calculate the basic package indicator and full set of demographic characteristics, and so were dropped in the demographic tables.

We considered socio-demographic factors with known association with the coverage indicators above. These include age of woman at time of delivery, household wealth, area of residence (urban or rural), education, parity, and child marriage. We defined child marriage using women’s age at first marriage or union. Women who were currently married or in union and less than 18 years of age, as well as women who were 18 or older whose age at first marriage or union was less than 18, were classified as child brides.

### Statistical analysis

We described the level of the coverage indicators, disaggregated by age groups, considering adolescents aged 15-19 years old and older women aged 20-49. Point estimates and 95% confidence intervals were computed. We ran a multilevel logistic regression model with random effects fitted for country in pooled data set, to explore the association between the receipt of the basic package of maternal health care and age group, controlling for socio-demographic factors. We exponentiated the coefficients and confidence intervals to get odds ratios for each fixed effects covariate, and examined between country variance from the random effects results.

All results were weighted and adjusted to account for survey multi-stage sampling design. We conducted all analyses in R version 3.3.1 (Foundation for Statistical Computing, Vienna, Austria).

## RESULTS

### Descriptive analysis

#### Characteristics of the study population

Across the 22 countries included in the analysis, 22 135 adolescents had a live birth in the two years preceding the survey. Out of this total 1806 (9.3%) received the basic package of maternal health care. Among the adolescents in the analysis 46% were among the poorest two quintiles and 32% were among the richest two quintiles. In terms of residence, 69% live in urban areas and 31% in rural areas. In terms of education achievement, 28% had no education, 40% started primary education and 30% started secondary education (**Table 2**).

**Table 2:** Demographic table by age of woman at time of delivery and receiving basic maternal health care package*

Covariates	Age group 15-19						Age group 20-49					
**Received basic package**						**Received basic package**					
**Yes**		**No**		**Total**		**Yes**		**No**		**Total**	
**N**	**%**	**N**	**%**	**N**	**%**	**N**	**%**	**N**	**%**	**N**	
**Household wealth:**												
Poorest	275	15.2	4059	24.0	4334	23.1	960	12.7	15 167	22.6	16 127	21.6
Poorer	329	18.2	3947	23.3	4276	22.8	1076	14.3	14 712	21.9	15 788	21.1
Middle	409	22.6	3761	22.2	4170	22.3	1369	18.2	13 869	20.7	15 238	20.4
Richer	432	23.9	3109	18.4	3541	18.9	1824	24.2	12 706	18.9	14 530	19.5
Richest	361	20.0	2054	12.1	2415	12.9	2305	30.6	10 705	15.9	13 010	17.4
**Area of residence:**												
Urban	1008	55.8	11 963	70.7	12 971	69.2	3661	48.6	46 114	68.7	49 775	66.6
Rural	798	44.2	4967	29.3	5765	30.8	3873	51.4	21 045	31.3	24 918	33.4
**Highest level of education:**												
No education	333	18.4	4998	29.5	5331	28.5	2118	28.1	28 102	41.9	30 220	40.5
Started primary education	680	37.7	6950	41.1	7630	40.7	2257	30.0	23 068	34.4	25 325	33.9
Started secondary education/higher	793	43.9	4981	29.4	5774	30.8	3156	41.9	15 978	23.8	19 134	25.6
**First time mother:**												
No	497	27.5	5584	33.0	6081	32.5	6307	83.7	61 108	91.0	67 415	90.3
Yes	1309	72.5	11 346	67.0	12 655	67.5	1227	16.3	6051	9.0	7278	9.7
**Child marriage:**												
No	863	47.8	6370	37.6	7233	38.6	4869	64.6	35 891	53.4	40 760	54.6
Yes	943	52.2	10 560	62.4	11 503	61.4	2665	35.4	31 268	46.6	33 933	45.4
**Total**	**1806**		**16** **930**		**18** **736**	**100.0**	**7534**		**67** **159**		**74** **693**	**100.0**

*The number of adolescents in the whole study is 22 135. Of those, 3399 are missing data to accurately calculate the basic package indicator, and so were dropped in the demographic tables. Source: authors’ analysis using data from DHS and MICS surveys included in the analysis.

#### Service use and practices across the maternal health continuum

##### Coverage of basic maternal health care indicators

Among adolescent mothers, the median coverage for at least 4 ANC visits was estimated at 50.6% (95% CI = 47.1%-54.2%), 40.9% (95% CI = 36.5%-45.4%) for specific routine services (blood test, blood pressure measurement, urine testing and neonatal tetanus vaccination), 65% (95% CI = 61.5%-68.9%) had a skilled attendant assisting the delivery of their last child and 45.7%(95% CI = 41.3%-50.2%) initiated breastfeeding within the first hour after delivery. However, only 9.3% (95% CI = 6.7%-11.7%) of them received the full set of all these interventions or services which define the basic package of maternal health care (Table S1 in the **Online Supplementary Document**).

Coverage of the other maternal health indicators not in the package was also low. The median percentage of adolescents aged 15-19 who reported having demand for family planning satisfied was 25.6% (95% CI = 20.6%-30.8%). The median percentage of adolescents who started ANC during their first trimester of pregnancy was only 24.9% (95% CI = 20.3%-29.5%) and only 3.5% (95% CI = 1.9%- 4.9%) had eight or more antenatal care contacts.

There was substantial cross-country variation across indicators included in this analysis. Country specific coverage of additional maternal health indicators are included in Table S1 in the **Online Supplementary Document****.**

**Figure 1:**
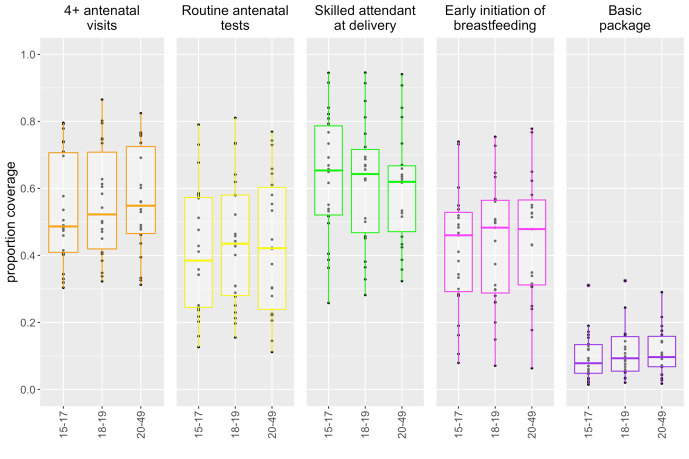
Coverage of basic maternal health care package across all 22 countries for all age groups younger adolescents (15-17 years), older adolescent (18-19 years) and older women (20-49 years). Figure presents the interquartile ranges of the coverage of the basic package of essential maternal care among 3 age groups: 15-17, 15-19 and 20-49. The dots represent country-level estimates, while the box plots represent the median, interquartile range, and minimum and maximum values across countries. The basic package is composed of 4 main interventions: 4+ antenatal care visits; ANC care (receipt of routine tests such as urine and blood test, blood pressure measured, tetanus toxoid injection); skilled attendant at time of delivery; and early initiation of breastfeeding. The denominator for the components of the basic package in this figure is women with live birth in last 2 years (n = 22 135 adolescents). Source: authors’ analysis using data from DHS and MICS surveys included in the analysis.

**Figure 1** displays boxplots of the proportion of women who received each intervention included in the basic package, stratified in three 3 age groups: adolescents 15-17, 18-19 and older women 20-49. The dots represent country-level proportions, while the boxplots show the median, interquartile range, minimum and maximum values across countries. Median coverage of indicators included in the basic package is low across the board. Younger adolescents have the lowest median coverage value of three out of the four components of the basic package except for skilled birth attendant. The co-coverage of the four indicators included in the basic package is extremely low and similar across the three age groups.

**Figure 2:**
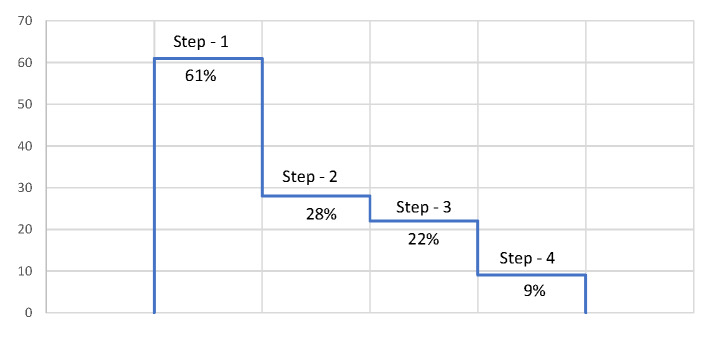
Cascade of loss of coverage (median across countries) from ANC4 to early initiation of breastfeeding among adolescent mothers 15-19. The basic package is composed of 4 interventions, which are differentially limiting among adolescents by country. Each dot represents the coverage in each of the countries in the analysis. Step 1 shows the proportion who received 4+ antenatal visits; Step 2 shows the proportion of adolescents who received both 4+ antenatal visits and routine antenatal tests (urine test, blood test, blood pressure measurement, and tetanus toxoid injection); Step 3 shows the proportion who received 4+ antenatal visits, routine tests, and had a skilled attendant at delivery; Step 4 shows the proportion who received 4+ antenatal visits, routine tests, had a skilled attendant at delivery, and initiated breastfeeding within the first hour after birth. Source: authors’ analysis using data from DHS and MICS surveys included in the analysis.

In order to understand the source of the low coverage values among adolescents that we observed for the basic package, we visualized the component indicators in a cascade with each step adding one indicator at a time, starting from ANC4 (**Figure 2**). The cascade assumes a continuum of care from ANC4 to early initiation of breastfeeding (EIBF) although the receipt of the ANC content interventions may not necessarily require four ANC contacts. However, the cascade shows where adolescents are more likely to drop off on the continuum between ANC and EIBF. Starting with the ANC4 as it is the component that had highest coverage, across countries, the coverage drops as each of the components is added reaching median levels below 10%. The largest drop was in the receipt of ANC content interventions. The median coverage dropped from 61% for ANC4+ to 28% for ANC4 and ANC content interventions. The next drop is in the early initiation of breastfeeding. This size of the drop is however variable across countries (Table S2 in the **Online Supplementary Document**). For a country like Congo the drop went from 77% ANC4+ to 46% when ANC content is added. Coverage remained mostly unchanged when skilled birth at delivery was included successively. Additional drop was observed when early initiation of breastfeeding was included. This pattern was observed in many countries in the analysis.

**Figure 3:**
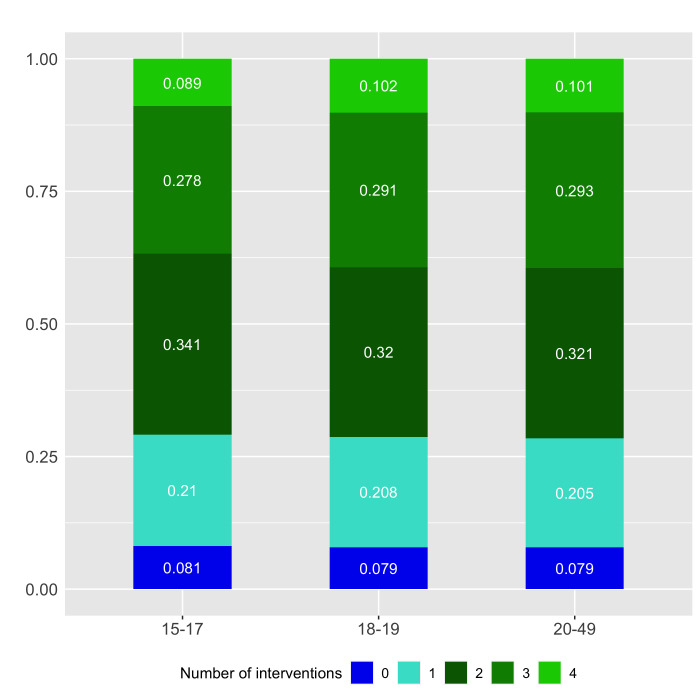
Co-coverage of basic maternal health care interventions and services. Distribution of number of interventions among women whose basic package components can be evaluated N = 18 736. Interventions include any combination of the following interventions: receiving 4+ antenatal visits; receiving routine antenatal tests (urine test, blood test, blood pressure measurement, and tetanus toxoid injection); having had a skilled attendant at delivery; having initiated breastfeeding within the first hour after birth. Source: authors’ analysis using data from DHS and MICS surveys included in the analysis.

**Figure 3** presents the co-coverage of the four indicators included in the basic package of maternal health care by the number of interventions or services that women across three age groups received. For 1, 2, 3 interventions or services, it refers to any combination of the 4 included in the basic package. The percentage distribution of each count is presented by age of the mother at time of delivery (15-17, 18-19 and 20-49). On average, less than 10% of women across the three age groups received zero maternal health interventions or services and 21% received at least one. Between 32 and 34 percent of women received any two interventions or services and between 28 and 29% received any three. About 36% of younger adolescents compared to 39% of older adolescents and adult women received three or more interventions or service contacts. However, only 9 to 10% of women across all age groups received all four basic maternal health interventions or services.

### Factors associated with receiving the basic maternal health package

To better understand the reasons for poor continuity of maternal care, we examined available social and economic factors which could potentially be associated with receiving the basic maternal health care package, namely age of woman at time of delivery, household wealth, area of residence, education, parity, and child marriage. Higher household wealth, urban residence, and higher educational achievement, were positively associated with receiving the basic maternal health package. On the other hand, younger age and having been a child bride were negatively associated with receiving the basic maternal health package. To be included in the inferential analysis, individuals had to have data for each of the components of the basic maternal health package outcome as well as each covariate, reducing the sample for the subsequent pooled analysis across all age groups to 18 736 women whose most recent birth was when they were ages 15-19 and 74 693 women 20-49.

**Figure 4:**
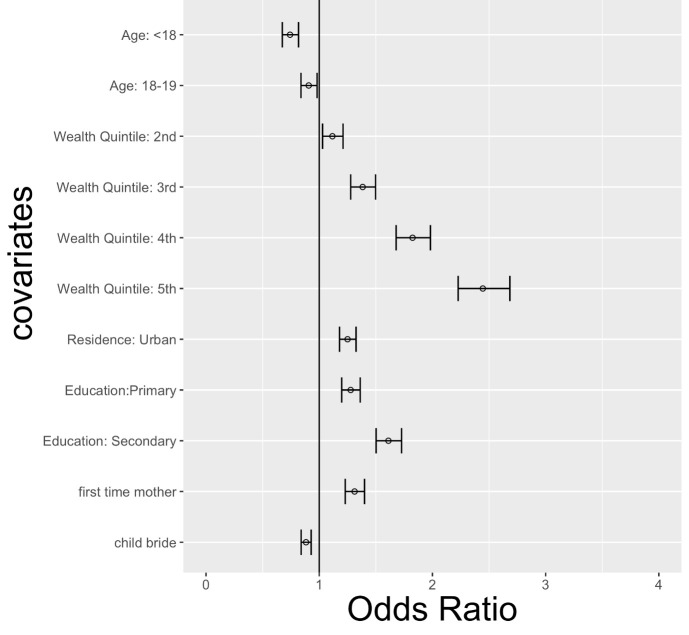
Odds ratios of the determinants of receiving the basic e package. Adjusted odds ratio (aOR): odds ratio adjusted for all other variables reported in the table. Reference categories: For age of woman at time of delivery: age 20-49; for wealth quintiles: 1^st^ quintile; for area of residence: is rural; for education: no education; for first time mother: not being a first time mother; and for child marriage: not having experience child marriage. Source: authors’ analysis using data from DHS and MICS surveys included in the analysis.

As presented in **Table 3** and **Figure 4**, women in the highest household wealth quintile had 2.44 times higher odds (95% CI = 2.23-2.68) of receiving the basic maternal care package compared to women in the lowest household wealth quintile. Living in an urban area was associated with 1.25 times greater odds (95% CI = 1.18-1.33) of receiving the basic maternal health care package and starting primary education and secondary education were associated with 1.28 (95% CI = 1.20-1.36) and 1.61 (95% CI = 1.50-1.73) times greater odds of receiving the basic package compared to women with no education. In contrast, adolescent girls aged 15-17 and 18-19 had lower odds of receiving the basic package as compared to women 20-49. The younger group of mothers had 26% lower odds (OR = 0.74, 95% CI = 0.67-0.82) and adolescent girls 18-19 had 9% lower odds (OR = 0.91, 95% CI = 0.84-0.98) of receiving the basic package as compared to women 20-49 years of age. Women whose first marriage occurred before the age of 18 years had 12% lower odds of receiving the basic package (OR = 0.88, 95% CI = 0.84-0.93). This is consistent with recent findings in the literature in which girl child marriage has been associated with increased fertility and reduced modern family planning, reduced antenatal care, and lower levels of safe delivery [25-27].

**Table 3:** Determinants of receiving the basic maternal health care package*

	Adjusted†	
	**aOR**† **(95% CI)**	***P*-value**
Age (years) of woman at time of delivery:		
**15-17**	0.74 (0.67, 0.82)	<0.001
**18-19**	0.91 (0.84, 0.98)	<0.001
**20-49**	reference	
Wealth quintile:		
**Poorest**	reference	
**Poorer**	1.12 (1.03, 1.21)	<0.001
**Middle**	1.38 (1.28, 1.50)	<0.001
**Richer**	1.82 (1.68, 1.98)	<0.001
**Richest**	2.44 (2.23, 2.68)	<0.001
Area of residence:		
**Rural**	reference	
**Urban**	1.25 (1.18, 1.33)	<0.001
Highest level of education:		
**No education**	reference	
**Started primary education**	1.28 (1.20, 1.36)	<0.001
**Started secondary education/higher**	1.61 (1.50, 1.73)	<0.001
First time mother:		
**No**	reference	
**Yes**	1.31 (1.23, 1.40)	<0.001
Child marriage:		
**No**	reference	
**Yes**	0.88 (0.84, 0.93)	<0.001

aOR – adjusted odds ratio, CI – confidence interval

*We fit a logistic regression model with fixed effects of age, wealth, residence, education, parity, and child marriage, and random effects for countries. Odds ratios for fixed effects are shown here. Women who did not have data for all covariates were excluded from this analysis. Odds overlapping with 1 do not differ statistically from the reference group, while ranges that fall below one decrease the odds of getting the basic package, and covariates with ranges higher than one increase the odds of getting the basic package. Source: authors’ analysis using data from DHS and MICS surveys included in the analysis. Odds ratios (OR): unadjusted associations between the variable and the outcome.

†Adjusted odds ratio (aOR): odds ratio adjusted for all other variables reported in the table.

Results from our random effects model also suggest that the relationship between various socioeconomic indicators and access to the basic package of maternal care varies by country. The variance for the random effect of country tells us how much of the covariate variance is explained by differences between countries. The among-country variance, 0.56, is larger than the magnitude of all of the treatment effects, except for the intercept (-3.04) and the fourth and fifth wealth quintiles (0.60, and 0.89, respectively), and this may reflect differences between countries in the norms, policies, and legislation pertaining to adolescent pregnancy and adolescents’ access to sexual and reproductive health services responsive to their needs.

## DISCUSSION

We examined coverage of key reproductive and maternal health services and determinants of receiving a basic package of maternal care among adolescents and older women in 22 sub-Saharan African countries with the highest adolescent birth rates in the world. Overall, the findings showed that even basic, yet critical services along and across the maternal health continuum, are not all reaching most women and particularly adolescents: Across these countries, the co-coverage of the four indicators included in the basic package of maternal services was only 9.3% (95% CI = 6.7%-11.7%) of adolescents who delivered in the two years preceding the surveys This co-coverage proportion ranges from 2% (95% CI = 1%-3.1%) of adolescents in Mali to 31.7% (95% CI = 26.7%-36.7%) in Liberia. Although coverage of many maternal health services has improved over the past two decades, progress towards increasing access to adolescent responsive maternal health services must also be accelerated in order to meet SDG aims of achieving universal coverage of maternal care for all women, including adolescent mothers. This is particularly important given the projected increase in adolescent births over the coming decade. An estimated 12.8 million adolescent girls have an unmet need for family planning in low- and middle-income countries, and this is projected to increase to 15 million by 2030 [28]. As part of efforts to reduce adolescent birth rates and improve maternal outcomes, improving knowledge of, access to and use of family planning services are essential. Available data on levels and trends of family planning indicators, including demand for family planning satisfied, contraceptive use and met need for spacing, indicate that adolescents do not have enough access to modern contraceptive methods [29-31]. These findings are consistent with the results in this analysis which show insufficient coverage of demand for family planning satisfied with modern methods: median of 25.6% (95C%CI: 20.6%-30.8%) among adolescents 15-19 years of age.

Our continuum of care approach with a co-coverage measure of four indicators in the basic package of maternal health services allowed us to uncover a surprisingly low co-coverage rate. To maximize improvements in maternal and newborn health outcomes among adolescents, progress must not only be measured at individual points of care, but across the continuum of antenatal, delivery and postnatal care [32]. We found that many women are not receiving all ANC basic content interventions which was most responsible for most of the drop in the co-coverage of the four interventions/service contract included in the basic package. This finding is indicative of the low quality of antenatal care receive by adolescents and adult women in these countries, where adolescent fertility is highest. Our findings suggest women of higher household wealth, higher educational attainment, and living in urban areas were more likely to receive this basic maternal care. In contrast, adolescent mothers and the ones who experienced child marriage were less likely to receive the basic package. The associations between women’s vulnerability and lower use of the basic maternal care package suggests that there is particular need to better understand and specifically address the barriers to continuity and quality of maternal care faced by the most disadvantaged women.

While improving access to these services by adolescents is essential, a critical strategy remains in addressing the prevalence of child marriage practices that lead to premature childbearing. Other strategies to be considered for reducing adolescent birth rate include addressing discriminatory gender and social norms as well as economic inequality, so that adolescent girls have the same opportunities as boys to be educated and to reach their full potential [33].

An essential component to increasing the chances that women receive all of the recommended maternal health services during pregnancy is ensuring that they make the minimum recommended number of ANC contacts [34]. For a positive pregnancy experience, WHO recommends that many of the interventions be initiated during the first trimester of pregnancy. Therefore, it is critical to develop strategies to facilitate earlier ANC initiation among pregnant adolescents [20].

Given that the majority of maternal deaths are preventable with routine, lifesaving interventions and an estimated one third of maternal deaths are due to complications that arise during labor, childbirth, and the immediate postpartum period, the large proportions of adolescent mothers delivering under sub-optimal conditions is contributing to poor maternal outcomes in countries with high adolescent birth rates [24]. Efforts to accelerate progress towards improved maternal health by 2030 must therefore focus both on improving appropriate service-seeking behaviors, as well as ensuring that all adolescents receive high quality, respectful care responding to their needs when they seek maternity care.

As immediate breastfeeding initiation is associated with improved newborn survival, long-term breastfeeding practices, child outcomes, health outcomes for the mother, and interventions to improve quality of maternal care, must include training on breastfeeding counseling [35].

Information on country adoption of legislation permitting adolescents to access contraceptive services without parental or spousal consent also shows that many countries with high adolescent fertility are also those with low adoption of such legislation. For instance, out of the 22 countries in the analysis, only eight countries have a policy in place for contraceptive services for adolescents without spousal or parental consent [31]. Similarly, many countries with high adolescent birth rates tend to have weak enforcement of or no introduction of legislation on the minimum age of marriage [31]. Also, gender and social norms play a key role in the tendency towards child marriage and early childbearing. For instance, these practices vary across countries in the Sahel region and countries in the coastal areas of West Africa. Additional research is therefore necessary to better understand the context-specific barriers to women’s access to essential and quality maternal care, and to develop effective interventions relevant for different contexts and the difference in patterns brought by country or region-specific social and gender norms.

This study has some limitations. As this is a secondary analysis of DHS and MICS data, data availability played an important role in shaping the study design. This affected both the countries and indicators included in the analysis. For instance, although interventions such as HIV testing, iron supplementation, breastfeeding counseling, deworming, and intermittent preventive treatment of malaria are all important components of antenatal care, we were unable to include them in our analyses due to lack of availability of data for these indicators across all of the study countries. Given the measurement issues and data availability of the postnatal care indicators, early initiation of breastfeeding was used as a proxy of postnatal care in the basic package of maternal health services. Additionally, all of the outcomes in the study rely on self-reported survey data, which may be subject to recall bias specifically in regard to women’s ability to correctly recall specific check-ups during the antenatal period or intrapartum period. Despite these limitations, this study is the first one analyzing a basic package of services using nationally representative data sets in 22 countries with a sample size of over 18 000 adolescents in sub-Saharan African countries with high childbearing among adolescents. The conclusions therefore are generalizable to other countries with similar characteristics. Our study provides useful insights into maternal health service use and content of care among adolescent girls and women in countries with high adolescent birth rates.

## CONCLUSIONS

Our findings demonstrate that the coverage of basic maternal health services for adolescent mothers 15-19 is insufficient in the countries with the highest adolescent birth rates in the world. Drivers of poor maternal health outcomes are multi-faceted and may vary across and within countries. Addressing the specific needs of adolescents, however, is of critical importance, especially in sub-Saharan Africa where adolescent births are expected to increase in the coming decades. Reducing the burden of maternal mortality will therefore require an integrated, comprehensive approach to ensure that reproductive and adolescent responsive health services are made available, accessible, and acceptable to adolescent girls and young women in need at all points throughout the reproductive, maternal, and child health continuum.

## Additional material

Online Supplementary Document
